# Beyond Conventional Treatments: The Role of Complementary Therapies in Head and Neck Cancer

**DOI:** 10.3390/cancers17081269

**Published:** 2025-04-09

**Authors:** Barbara Verro, Simona Fiumara, Giuseppe Saraniti, Gaetano Ottoveggio, Carmelo Saraniti

**Affiliations:** 1 Division of Otorhinolaryngology, Department of Biomedicine, Neuroscience and Advanced Diagnostic, University of Palermo, 90127 Palermo, Italy; carmelo.saraniti@unipa.it; 2 Division of Speech Therapy, Private Practice, 90100 Palermo, Italy; simona.fiumara89@gmail.com; 3 Department of Clinical Pathology, Ospedale Buccheri La Ferla, 90123 Palermo, Italy; saraniti.giuseppe@fbfpa.it; 4 Unit of Anesthesia Analgesia Intensive Care and Emergency, Department of Precision Medicine in Medical, Surgical and Critical Care University Hospital Policlinico Paolo Giaccone, 90127 Palermo, Italy; gaetano.ottoveggio@policlinico.pa.it

**Keywords:** head and neck cancer, complementary therapy, acupuncture, herbal medicine, cannabinoids, traditional Chinese medicine, mind-body therapies

## Abstract

Background/Objectives: Head and neck cancer is one of the most common cancers globally, with high mortality and significant treatment-related side effects. Conventional therapies, including surgery, radiotherapy, and chemotherapy, have improved survival but often have serious consequences for patients’ quality of life. For this reason, there is growing interest in complementary therapies such as acupuncture, herbal medicine, cannabinoids, traditional Chinese medicine, and mind-body therapies. Methods: This review was conducted through a systematic analysis of the scientific literature available on PubMed and Scopus, selecting studies about the use of alternative therapies in patients with head and neck cancer according to strict criteria. Results: Acupuncture has shown benefits in the management of xerostomia and dysphagia, while some herbal medicines have shown potential anticancer effects, although with limitations related to bioavailability. Vitamins and antioxidants showed mixed results: some studies suggest a protective effect, while others report a possible increased risk of cancer progression. Cannabinoids are a controversial topic, with possible palliative benefits but also a higher risk of head and neck cancer. Traditional Chinese medicine and mind-body therapies, such as yoga, have shown positive effects on patients’ well-being, although their direct impact on cancer progression remains uncertain. Conclusions: Alternative therapies could be a useful support in managing symptoms and improving the quality of life patients with head and neck cancer. However, solid scientific evidence on their effectiveness and safety is still lacking. Rigorous clinical studies are needed to assess their therapeutic potential and define a safe integration into multidisciplinary cancer management.

## Simple Summary

Head and neck cancer is a widespread disease with serious consequences for patients’ quality of life. Conventional treatments, such as surgery, radiation, and chemotherapy, can be effective but often cause significant side effects. For this reason, many patients seek complementary therapies to relieve symptoms and improve well-being. This review looks at several alternative therapies, including acupuncture, herbal medicine, vitamins, cannabinoids, traditional Chinese medicine, and mind-body therapies. Some of these strategies, such as acupuncture for xerostomia or herbal therapy for cancer support, have shown promising results, while others raise doubts about their safety and effectiveness. Although alternative therapies may offer benefits, definitive scientific evidence is still lacking. This research highlights the importance of rigorous clinical trials to assess their real impact and ensure that they are used safely, as a support and not as a substitute for standard care.

## 1. Introduction

Head and neck cancers (HNCs) are among the most prevalent malignancies worldwide, ranking as the seventh most common type of cancer. HNCs encompass several anatomical sites, including the oral cavity, oropharynx, hypopharynx, larynx, salivary glands, nasopharynx, nasal cavity, sinus cavities, and ear [1]. The majority of these tumors are squamous cell carcinoma (SCC) [2]. Currently, HNCs account for over 660,000 new cases diagnosed and 325,000 deaths annually [3]. According to the 2020 Global Cancer Observatory (GLOBOCAN) (https://www.uicc.org/news/globocan-2020-global-cancer-data, accessed on 8 January 2025), the incidence of HNCs will increase by 30% annually by 2030 [3]. This increase is particularly notable for oral cancer (OC) in Asia, primarily due to betel leaf chewing, and oropharyngeal cancer (OPC) in the United States of America (USA) and Europe, due to the increased spread of Human PapillomaVirus (HPV) infection [4,5,6]. Men are more affected than women, with a male-to-female ratio of 4:1 [7]. The main risk factors include tobacco smoking and alcohol consumption, followed by HPV infection, occupational or environmental exposure to carcinogens, diet, cannabis use, poor oral health, and genetic susceptibility to develop this type of cancer [8,9,10,11,12]. The prognosis of HNCs depends on several factors, including the tumor’s anatomical site, tumor stage (according to the tumor-node-metastasis TNM stage system), histology, as well as patient health status, considering age, risk factors, performance status, and socioeconomic conditions [13,14]. To date, the therapeutic strategies include surgery, radiotherapy (RT) and/or chemotherapy (CT), and immunotherapy [15]. However, despite advancements in research and treatment development, the five-year survival rate for HNCs remains disappointingly low. Several critical factors contribute to this outcome, including late diagnosis, which limits effective intervention; metastases and relapses, which complicate disease management; and resistance to therapies, which limits the effectiveness of available treatments and makes it necessary to develop new ones [16]. Moreover, although often effective in controlling the disease, conventional therapies often have severe side effects that significantly impact patients’ quality of life. These side effects may vary in severity and nature depending on the type of treatment and include both physical and psychological consequences. RT, a cornerstone treatment, is frequently associated with xerostomia, mucositis, and dysphagia [17,18,19,20]. CT can induce immunodeficiency, exposing patients to an increased risk of infections that can further complicate the course of the disease [21,22]. Surgery can lead to significant aesthetic and functional consequences, affecting self-esteem and social life [23,24,25,26]. In addition, one of the most distressing complications, associated with both the disease and its treatment, is the need for a tracheostomy, which has profound physical and psychological effects. 

Given these challenges, some patients, influenced by concerns over treatment side effects or in search of a definitive cure, turn to alternative therapies, sometimes even abandoning conventional treatments. However, many of these therapies lack robust scientific validation and are not endorsed by the medical community. 

Within the limits of the information available in the main databases of scientific research, this review aims to explore the alternative therapies used in HNC management, both as standalone approaches and in combination with conventional treatments. Their possible or presumed effectiveness will also be analyzed. The ultimate goal is to assess the benefits and limitations (“boon and bane”) of these therapies in order to ensure the best possible care for the patient while improving his quality of life.

## 2. Search Methodology and Data Analysis

The review was followed the Preferred Reporting Items for Systematic Reviews and Meta-Analyses (PRISMA) guidelines. The review is based on a bibliographical search conducted in major scientific databases, including PubMed (https://pubmed.ncbi.nlm.nih.gov, accessed on 7 January 2025) and Scopus (https://www.scopus.com, accessed on 7 January 2025), searching for the following keyword strings: head and neck cancer OR oral cancer OR laryngeal cancer OR pharyngeal cancer OR ear cancer OR paranasal sinus cancer OR nose cancer AND alternative therapy OR vitamins OR complementary therapy.

Inclusion criteria were: studies about head and neck cancer, both retrospective and prospective studies, and articles written in the English and/or Italian language.

Exclusion criteria were: case reports with less than 5 patients, studies not dealing with alternative therapies, studies about non-HNC, systematic reviews, and meta-analyses.

**Figure 1:**
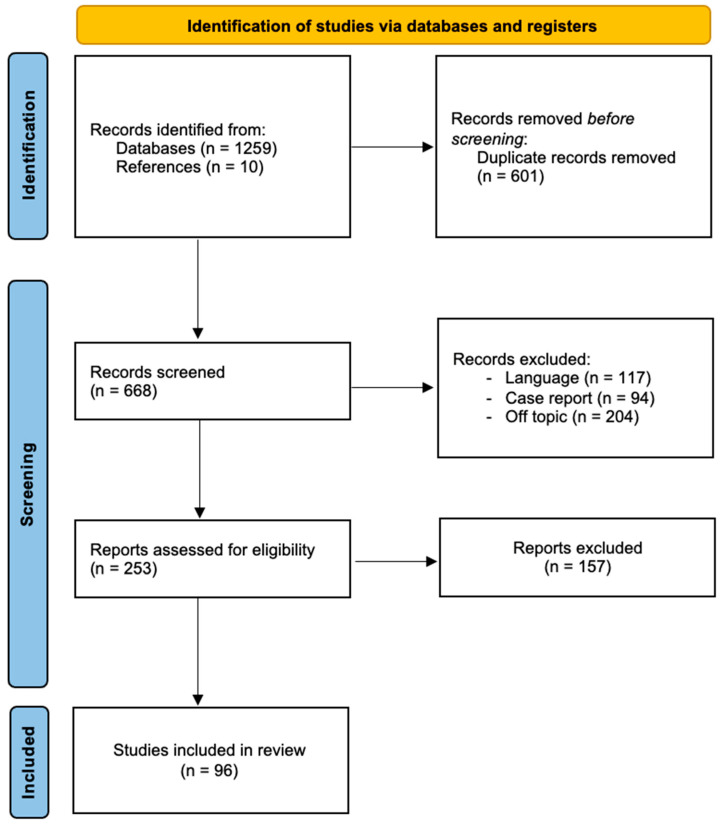
PRISMA 2020 flow diagram of the study selection process of the literature [27].

In total, 1259 articles were identified through the database searches. After removing 601 duplicates, two authors (B.V. and C.S.) screened 668 articles by reviewing their titles and abstracts, applying selection criteria such as language (117), case reports (94), and relevance (204). Following this, the same authors conducted a full-text analysis of 253 selected manuscripts, ultimately including 96 articles in this review, of which 10 were obtained from the reference lists of the selected studies (Figure 1) [27].

## 3. Complementary Therapies

### 3.1. Acupuncture

Acupuncture has been a cornerstone of Traditional Chinese Medicine for approximately 3000 years and has demonstrated safety and effectiveness in treating various conditions, including dysphagia [28]. In 2003, the World Health Organization (WHO) reported the efficacy of acupuncture based on clinical studies, highlighting the conditions for which acupuncture has demonstrated therapeutic benefits [29]. 

As previously written, RT causes xerostomia in about 50% of patients due to the high sensitivity of the salivary glands, with irreversible damage at high radiation doses (greater than 50 gray Gy) [30]. In 1993, Blom et al. demonstrated first that acupuncture improved vascularity near the parotid gland affected by Sjogren’s syndrome [31]. Based on this study, other research has been conducted to demonstrate the effectiveness of acupuncture in radiotherapy-induced xerostomia [32,33,34,35,36]. In particular, in their randomized clinical trial, Garcia et al. compared three types of xerostomia’s treatment: standard care control, sham (placebo) acupuncture, and true acupuncture [30]. Sham acupuncture consists of non-penetrating needles on ineffective locations, following the same scheme as the real acupuncture [37,38]. According to the literature, true acupuncture includes the following points: each ear (3 sites), chin (1 site), each forearm (1 site), each hand (1 site), and each leg (1 site) [39,40]. The authors demonstrated that after one year, patients in the true acupuncture group reported significantly lower xerostomia scores (Xerostomia Questionnaire) than in the standard cure group, with a statistically significant difference (*p* = 0.001) [30]. Meng et al. performed acupuncture three times a week during RT with the attempt to prevent xerostomia. This randomized controlled trial showed that patients who underwent true acupuncture scored significantly lower on the Xerostomia Questionnaire than the placebo control group (*p* = 0.006), from the third week of treatment and up to one month after the end of RT. Clinical improvements were maintained up to six months post-treatment, albeit reduced. However, no significant differences between the groups were observed in objective measurements of unstimulated or stimulated salivary flow [34]. Similarly, Cohen et al. conducted a randomized, blinded, controlled trial and reported that after 12 weeks, patients in the true acupuncture group reported a significant reduction in symptoms (Xerostomia Questionnaire and Functional Assessment of Cancer Therapy–General) compared to the standard care group; however, the difference between true acupuncture and placebo acupuncture did not reach statistical significance. In particular, at the beginning (weeks 8 and 12), the true acupuncture group had lower Xerostomia Questionnaire scores than the placebo acupuncture group. Though, in the long-term (week 26), that advantage had disappeared for true acupuncture [41].

Acupuncture has also proven to be both safe and effective in aiding the recovery of dysphagia following laryngectomy, as it supports the restoration of new synaptic connections [42]. Based on this assumption, in their experimental prospective study, Xuewei et al. demonstrated that acupuncture can directly activate the swallowing muscles while also stimulating the glossopharyngeal nerves, enhancing medulla oblongata excitability, and supporting the restoration of the swallowing reflex arc [43]. The authors underlined that the combination of acupuncture and swallowing rehabilitation exercises offers better results compared to individual therapies in the case of post-laryngectomy dysphagia. 

Furthermore, Ben-Arie et al. analyzed the effect of acupuncture on feeding intolerance after surgery (resection plus reconstruction) due to oral or hypopharyngeal cancer [44]. The authors conducted a single-blind study comparing the acupuncture group (acupoints specifically for indigestion) and the control group (acupoints not-specifically for indigestion): they found that the intervention group achieved the target energy expenditure earlier (*p* = 0.012), showed a higher caloric intake in the first post-operative week (*p* = 0.004), and required less prokinetics, like metoclopramide (*p* = 0.010), in comparison with the control group.

### 3.2. Phytotherapy

Over 70% of FDA-approved (Food and Drug Administration) anticancer drugs originate from natural plant-derived products, many of which have been used for centuries in traditional medicine to treat various diseases (e.g., the anticancer drug vinblastine, extracted from Vinca rosea, from the ayurvedic medicine) [45]. Today, it is well known that a diet rich in fruits and vegetables as well as herbal supplements, with known antioxidant action, can reduce the risk of developing cancer, especially oral cancer [46].

#### 3.2.1. Curcumin

Curcumin is a polyphenol extracted from the rhizome of the herb Curcuma longa, the turmeric. 

It results in effectiveness in managing the side effects of cancer treatment (chemoradiotherapy): reduction in oral mucositis, reduction in weight loss, and increased adherence to chemoradiotherapy. Indeed, patients treated with curcumin had a significantly lower incidence of severe mucositis than the control group (placebo), suggesting a protective effect of curcumin on oral epithelial cells (*p* < 0.001) [47,48]. A recent study by Martins et al. evaluated the efficacy of a mucoadhesive medicine (mouthwash) containing curcuminoids (20 mg/mL) and glycerinated extract (40%) from Bidens pilosa (FITOPROT), used twice per day, in the treatment of oral mucositis induced by RT and quality of life in patients affected by HNC [49]. The sample was divided into two groups: Group 1 received photobiomodulation (PBM) and a Preventive Oral Care Program (POCP), while Group 2 received PBM, POCP, and FITOPROT. This double-blind clinical trial showed no statistically significant differences between the two groups in the improvement of oral mucositis (Patient-Reported Oral Mucositis Symptom Scale). Moreover, both groups experienced an impairment in quality of life during RT by applying the University of Washington Quality of Life Questionnaire. Therefore, the study suggests that FITOPROT plus PBM and POCP does not provide additional benefits in controlling the severity of oral mucositis or stabilizing the quality of life in these patients. Furthermore, another randomized double-blinded placebo-controlled trial demonstrated that HNC patients who received capsules of curcumin (1 or 1.5 g/day) for six weeks during chemoradiotherapy showed less weight loss, suggesting that treatment helped preserve oral mucosa integrity and improve nutritional intake (*p* < 0.05) [50]. Consequently, the reduction in adverse effects is related to increased adherence and tolerance to chemoradiotherapy (*p* = 0.002) [48].

Palatty et al. conducted a prospective double-blinded randomized study that evaluated the effectiveness of a cream based on sandalwood and turmeric oil in the prevention and treatment of radiodermatitis in 50 HNC patients undergoing external beam RT [51]. Radiodermatitis can result from either direct radiation impact on cells or secondary chemical reactions and inflammations that aggravate the problem. Prophylactic application of the cream began on the first day and was continued daily for up to two weeks after completion of RT. Both creams were applied to the irradiated area five times a day. The study demonstrated that the use of this cream– before and after RT– significantly delayed and reduced the severity of radiodermatitis (according to Radiation Therapy Oncology Group RTOG guidelines) compared to the control group (Johnson’s baby oil) (*p* < 0.01). Thus, this cream, which has been used in India for 40 years, could be a safe and effective option to prevent radiodermatitis, thanks to the wound-healing and skin-protective properties of sandalwood and turmeric oil. Both ingredients have antioxidant, anti-inflammatory, and antimicrobial effects, which help protect the skin from radiation-induced damage, reduce inflammation, and promote faster healing.

Curcumin is also useful in treating symptoms related to cancer and its therapy, like depression and neuropathic pain. Depression is quite common in cancer patients. Studies found higher IL-6 and TNF serum levels in depressed cancer patients than in non-depressed ones and healthy people [52,53]. Experiments on stressed rats showed that curcumin can reduce serum expression levels of these two cytokines [54] and inhibit monoamine oxidase (MAO) activity [55]. Neuropathic pain may be due to higher expression of inflammatory cytokines, like Tumor Necrosis Factor (TNF). Indeed, a laboratory study on mice showed that curcumin can decrease this pain by reducing TNF expression and promoting the release of nitric oxide [56,57].

The safe and therapeutic (anti-cancer) curcumin dose is not well established yet, ranging from 10 to 12 g of oral daily intake [58,59]. However, it is characterized by poor bioavailability due to important first-pass metabolism [60]. Because of this, curcumin analogues and nanoformulations have been developed to improve their bioavailability as well as their potential biological effects in vitro and in vivo experiments [61,62,63]. Nevertheless, Mazzarino et al. reported a greater cytotoxic power of free curcumin in comparison with curcumin nanoformulations [64]. 

Several studies also demonstrated that curcumin has anticancer therapeutic potential: breast cancer, HNC, melanoma, and gastrointestinal cancer [56]. In particular, in the case of oral cancer, in vitro studies showed that curcumin can hinder cancer cell growth by inhibiting the progression of the S/G2M phase [65,66], enhance the cytochrome P450 expression, so reducing the risk of developing cancer [67], promote apoptosis of oral cancer cells by inhibiting cyclooxygenase-2 (COX-2), and increase the RT sensitivity [68,69]. A study conducted on hamsters found that curcumin can reduce the incidence of oral SCC as well as decrease the cell proliferation in benign and pre-malignant oral diseases (e.g., papilloma, dysplasia) [65]. A recent paper by Zhao et al. reported that curcumin has anti-cancer effects through various mechanisms, including: (1) cell cycle arrest by stopping the progression of cancer cells; (2) induction of apoptosis, both intrinsic and extrinsic pathways; (3) production of reactive oxygen species (ROS) by causing oxidative stress in cancer cells; (4) inhibition of the epithelium-mesenchymal transition (EMT): reducing the capacity of cancer cells to invade and metastasize; (5) modulation of the tumor microenvironment (TME) by affecting cancer-associated fibroblasts (CAFs) and immunity, both innate (e.g., dendritic cells, monocytes, mast cells) and adaptive (e.g., T-cells) [70,71,72,73,74]. The administration of curcumin in combination with RT and CT (e.g., cisplatin and 5-fluorouracil) was found to have a greater effect than the single treatments [75].

#### 3.2.2. Black Raspberry

The study on the anticancer effects of black raspberry has highlighted its potential in preventing and treating oral carcinoma through in vitro and in vivo studies on animal models. One of the first studies to investigate the anticancer effects of black raspberry was conducted by Seeram et al. [76], who tested a methanolic extract of the fruit on oral cancer cells CAL-27. The results showed the reduction in cell proliferation without identifying the mechanism of action. Subsequently, Nedungadi et al. used a combined ethanolic and aqueous extract of the same fruit, also on the CAL-27 cell line, demonstrating that this treatment led to a suppression of tumor growth and an increase in cancer cell apoptosis. Moreover, the treatment reduced the expression of PCNA (Proliferating Cell Nuclear Antigen) and Bcl-2, two proteins involved in cell proliferation and apoptosis resistance, respectively. Finally, an increase in the cancer suppressor gene CDKN1A has been observed [77]. To assess the potential of black raspberry in modulating tumor growth in living organisms, several animal model studies have been conducted. In particular, an internally paired study by Warner et al. [78] investigated the effects of treatment with black raspberry powder on male hamsters, in which oral cancer had been chemically induced with the carcinogen 7,12-Dimethylbenzathracene (DMBA). Hamsters treated with topical black raspberry for a prolonged period (three times/week for six weeks) showed a decrease in the number of tumors and their incidence, as well as a reduction in tumor cell proliferation. In addition, after only two weeks of treatment, upregulation of the tumor suppressor gene Rb1 was observed, indicating a possible protective effect of black raspberry in the initial stage of carcinogenesis. Another placebo-controlled study, conducted by Oghumu et al. on F344 rats affected by oral cancer, confirmed these findings by administering a diet containing 5% or 10% black raspberries for six weeks [79]. 

A compound of black raspberries and plants with brightly colored flowers and fruits is anthocyanin, a water-soluble colorant belonging to the family of flavonoids. This flavonoid has an antioxidant and anti-tumor action, inhibiting tumor growth [80]. In the case of oral cancer, the anthocyanin gel can be used with local effects, reducing systemic side effects. Indeed, the presence of saliva (pH 6.5) promotes the absorption of this flavonoid and therefore its antioxidant activity [81].

#### 3.2.3. Cranberry

Studies indicate that cranberry could have a promising action against oral carcinoma, thanks to its ability to reduce tumor proliferation and promote apoptosis. One of the first in vitro studies on this topic was conducted by Seeram et al., who tested a methanolic cranberry extract on the oral carcinoma cell line CAL-27. Treatment led to a reduction in cell proliferation, although the study did not specify the molecular mechanisms underlying this effect [76]. Subsequently, Chatelain et al. conducted a study by using a commercial cranberry extract to treat two different oral cancer cell lines, CAL-27 and SCC-25. The authors demonstrated not only a decrease in cell proliferation but also a reduction in adhesion and viability of cancer cells, suggesting that the extract could compromise their ability to survive and spread. Molecular mechanisms analysis revealed increased levels of apoptotic proteins caspase-2 and caspase-8, a sign of increased activation of programmed cell death. In addition, in CAL-27 cells, treatment with cranberry has led to an increase in p53 expression, a cancer suppressor that regulates cell cycle and apoptosis [82].

#### 3.2.4. Green Tea

Green tea is another substance with anti-tumor properties thanks to the four types of polyphenols that it contains. Indeed, in oral cancer, the antioxidant activity of these polyphenols can inhibit cancer cell growth and promote their apoptosis as well as prevent gene mutation and thus the development of cancer itself [83]. In particular, Tsao et al. conducted a phase II placebo-controlled double-blinded trial and administered green tea extracts to 28 patients affected by high-risk oral premalignant lesions, comparing results with placebo. The patients were subdivided into four groups according to green tea extract dose (500, 750, or 1000 mg/m^2^) orally taken three times per day after meals for 12 weeks. The authors found a regression of lesions compared to the placebo group, the expression downregulation of some biomarkers associated with carcinogenesis, like cyclin D1 and stromal vascular endothelial growth factor. The few side effects, namely insomnia and nervousness, were nevertheless mild and tolerable [84]. An in vivo study by Li et al. examined the effects of green tea on oral carcinogenesis induced by DMBA in male hamsters. In this research, a 0.6% aqueous solution of green tea was applied directly to the oral mucosa of hamsters after treatment with DMBA for a period of 18 weeks. The results showed that treatment with green tea led to a reduction in the number and volume of tumors and an increase in apoptosis of dysplastic cells, suggesting a protective effect against oral cancer progression. However, the study did not identify the molecular mechanisms behind these effects [85]. These findings support the potential chemopreventive role of green tea in oral carcinoma, reinforcing interest in the use of natural compounds in the prevention and treatment of head and neck cancers.

#### 3.2.5. Tomato

This carotenoid, known for its antioxidant properties, plays a key role in reducing the incidence and progression of oral cancer through several biological mechanisms. 

Singh et al. conducted a randomized prospective placebo-controlled study of 58 patients affected by oral leukopenia treated with oral lycopene or placebo. The study showed that lycopene treatment led to a significant improvement in the reduction in leukoplakia lesions (*p* < 0.05). The clinical response to treatment was determined through two-dimensional measurement of lesions and photographic documentation. Histological evaluation, including classification of the degree of dysplasia, was performed in blind before and after treatment. Moreover, the authors found that the efficacy of lycopene may be dose-dependent: 8 mg per day is more effective than 4 mg, although even this last dose has shown significant clinical benefits in the treatment of oral leukoplakia, probably requiring a longer period of administration [86]. Also, Mayne et al. investigated the potential anti-tumoral role of lycopene, finding that patients with prior oral, pharynx, or larynx cancer and low levels of lycopene in the blood showed a higher risk of mortality (*p* < 0.05), suggesting a possible protective role of this carotenoid [87]. Ye et al. carried out a study on human HNC cell lines treated with different concentrations of lycopene. The authors investigated its possible role in tumor proliferation and invasion as well as the expression of proteins involved in tumor progression. They reported that lycopene can hinder tumor proliferation by inhibiting the MAPK and PI3K/AKT signaling pathways and promote cancer cell apoptosis by upregulating the pro-apoptotic caspase 3 and Bax expression [88].

In their randomized placebo-controlled studies, Chandra et al. [89] evaluated the effects of a lycopene-rich tomato acetone extract in an oral carcinogenesis model induced by DMBA in male Syrian hamsters. Animals treated with intragastric tomato extract three times per week showed a significant reduction in both the incidence and severity of oral mucosal tumors. This effect has been accompanied by an increase in the activity of key antioxidant enzymes such as glutathione peroxidase (GPx), superoxide dismutase (SOD), and catalase (CAT), not only in oral mucosa lesions but also in the liver and erythrocytes of these animals. A further study, conducted by Bhuvaneswari et al., confirmed these results, using seedless tomato paste. The treatment resulted in a significant reduction in tumor incidence as well as a reduced presence of dysplasia in oral mucosa cells exposed to DMBA. This effect occurred independently of the concentration of lycopene (2.5, 5, and 10 mg/kg) present in tomato paste, suggesting that the protective effect of tomato could be the result of a combination of more bioactive compounds than just lycopene. In addition, a significant increase in enzymatic activities of SOD, CAT, and GPx was observed, confirming the action of tomato in strengthening the antioxidant defenses [90]. 

Overall, these studies suggest that the consumption of tomato and tomatoes products could be an effective strategy in preventing oral cancer, due to their ability to modulate oxidative stress and promote the protection of healthy tissues. The action of lycopene, although central, could be enhanced by the presence of other bioactive compounds in tomatoes, making it a true functional food with a potentially significant impact on oral health and oncology.

#### 3.2.6. Soybean

Soybean is a legume with chemotherapeutic activity due to its content in isoflavones, with estrogen-like features. In particular, in 2001, Meyskens et al. studied the Bowman-Birk inhibitor (BBI), a natural protein derived from soy, in the prevention of the development of oral cancer. The study demonstrated that BBI can reduce the tumor size. This result suggests its role in preventing oral cancer progression by modulating the levels of serine proteases involved in disease progression [91]. Conversely, a double-blind, placebo-controlled phase II-B trial was conducted to investigate the anti-cancer power of BBI. This study did not find any significant difference between the BBI group (3 grams twice a day for six months) and the placebo group (3 grams twice a day for six months) in clinical response (*p* > 0.94), reduction in leukoplakia area (*p* > 0.75), histological degree of lesions (*p* > 0.88), and biomarkers analyzed (buccal-cell Neu protein, serum-Neu protein, protease activity) (*p* > 0.30) [92]. In conclusion, these results indicate that BBI, despite its good tolerability, did not show a clinically significant effect on oral leukoplakia compared to placebo. However, the authors reflect critically on their results, assuming that the choice of placebo may have influenced the outcome of the study. In fact, the authors have chosen as a placebo a product based on corn (Masa Harina), considered suitable for its apparent inertia, agreeableness, and physical similarity with BBI. However, this compound contains numerous micronutrients, including vitamins, antioxidants, and other potentially active elements. The authors themselves recognized that this placebo may have had a preventive effect on cancer, thus making it more difficult to interpret the actual effectiveness of the experimental treatment.

A phase II clinical trial was conducted at the University of Michigan in patients affected by primary or locally recurrent HNC [93]. The authors reported that neoadjuvant soybean isoflavone oral supplementation (300 mg/day for three weeks before surgery) can promote LINE-1 methylation in HNC tissue (epigenetic change), thus promoting DNA stability, where LINE-1 hypomethylation is an event of the early stages of carcinogenesis [94].

#### 3.2.7. Black Rice

Black rice has long been appreciated for its nutritional benefits, but in recent years research has also highlighted its anti-cancer potential, thanks to the presence of anthocyanins and other bioactive compounds. An in vitro study examined the effects of a black rice pericarp ethanolic extract on oral carcinoma cells CAL-27. The results showed a significant decrease in the proliferation and invasive capacity of cancer cells, suggesting that the compounds present in black rice may hinder oral cancer progression. The action of the extract seems to be linked to several mechanisms. First, treatment has shown the ability to limit the spread of cancer cells by reducing the expression of key proteins involved in migration and metastasis. Moreover, it affected the growth cycle of cancer cells, slowing their proliferation. Its anti-inflammatory effect was expressed by reducing the activity of NF-κ B, which promotes tumor progression. This has also led to a reduced production of degrading enzymes, responsible for the destruction of the extracellular matrix and therefore the ability of the tumor to be invasive. In addition, the treatment interfered with some signaling pathways that are crucial for cancer cells to survive, blocking proteins that promote tumor growth and expansion [95]. These results suggest that black rice could be useful in the prevention and treatment of oral cancer.

#### 3.2.8. Cinnamon

Chinese cinnamon is known for its many beneficial properties, including anti-inflammatory and anticancer effects. Yu et al. evaluated the efficacy of an ethanolic extract of Chinese cinnamon on several oral carcinoma cell lines (OC2, SCC-4, SAS, and SASVO3). The results showed that treatment led to a reduction in cell viability, with a decrease in the number of cancer cells. This effect was accompanied by an increase in apoptosis, as evidenced by the activation of caspase-3 and reduction in Bcl-2, a protein that helps cancer cells resist programmed cell death. In addition to apoptosis, the treatment also activated autophagy processes, suggesting a double mechanism through which cinnamon extract could affect cancer cells [96]. The efficacy of Chinese cinnamon in fighting oral carcinoma has also been supported by in vivo studies, such as that conducted by Ezzat et al. on hamsters with DMBA-induced oral carcinogenesis. In this randomized placebo-controlled study, animals treated with an aqueous extract of cinnamon bark (500 mg/kg for 16 weeks) showed a significant reduction in the growth of tumor lesions, with a lower incidence of necrosis and ulceration than the control group. Tissue analysis also revealed a reduction in differentiation cluster 3 (CD3) and platelet-derived growth factor (PDGF), suggesting a direct impact on the tumor microenvironment, probably through the modulation of the inflammatory and angiogenetic response [97].

#### 3.2.9. Garlic

Studies conducted on the action of garlic in contrast to oral carcinoma have revealed surprising results, confirming its potential as a chemotherapeutic agent. One of the first studies revealed that Golden Syrian hamsters treated with garlic showed slower tumor growth than controls, with smaller tumors, reduced cell proliferation, and fewer new blood vessels. In particular, the preventive administration of garlic appeared to have a significant protective effect by reducing the incidence of cancerous lesions [98]. These results were confirmed by the randomized placebo-controlled studies by Balasenthil et al., who deepened the molecular mechanisms underlying this action. It was observed that Syrian hamsters treated with garlic extract (250 mg^−1^/kg orally three times per week for 14 weeks) were not only free of malignant neoplasms but also showed an improvement in biological markers related to tumor progression. Levels of plasma antioxidants such as vitamin C, vitamin E, and glutathione were significantly increased, indicating a strengthening of the defenses against oxidative stress, one of the key factors in neoplastic transformation. Another important finding was the impact of garlic on the mechanisms of apoptosis in cancer cells. Treatment with garlic led to an increased expression of tissue transglutaminase, a protein involved in the activation of apoptosis, while the Bcl-2, which inhibits cell death and promotes cancer cell survival, was significantly reduced. This balance between survival and cell death suggests that garlic could not only prevent the onset of oral cancer but also counteract its progression [99,100,101,102].

#### 3.2.10. Grape

Studies on the effects of grape and its extracts in the treatment of oral carcinoma have shown promising results, suggesting a chemopreventive and therapeutic potential. Both grape seed and peel extracts have been shown to exert anti-cancer action through different cellular and molecular mechanisms. In vitro studies conducted on oral carcinoma cell lines, such as CAL-27, SCC-25, Ca9-22, and TCA8113, showed that exposure to grape seed extracts leads to a reduction in cell proliferation and adhesion, accompanied by an increase in apoptosis. This effect has been associated with the activation of several pro-apoptotic markers, including caspases-2, -3, -8, and the Bax protein [82,103]. A further study, conducted on tongue carcinoma cells (TCA8113) by Yang et al., showed that treatment with grape seed extract leads to reduced migration and invasion of cancer cells due to the suppression of the Akt/NF-κ B signaling pathway, as well as the decreased expression of MMP-2 and MMP-9 metalloproteinases, essential for the degradation of extracellular matrix and spread of malignant cells [104]. The in vivo studies confirmed what was observed in vitro, showing that the grape seed and peel extract is also effective in animal models. Shrotriya et al. showed that the integration of grape seed extract (0.2% weight/weight for eight weeks) into the diet of six mice with precancerous oral lesions reduced the progression of lesions, decreasing both the incidence and proliferation of cancer cells, in comparison to the placebo group [105]. These results suggest that grape extracts, thanks to their effects on numerous molecular mechanisms from the reduction in cell proliferation and adhesion to the induction of apoptosis and the modulation of inflammatory and oxidative processes may represent a promising chemopreventive and therapeutic approach against oral carcinoma.

#### 3.2.11. Pomegranate

Studies about the anti-cancer effects of pomegranate in oral carcinoma have revealed its ability to inhibit cell growth, reduce tumor invasion, and induce apoptosis in malignant cells. One of the most significant studies, conducted by Peng et al., showed that treatment with pomegranate extract reduced cell viability by more than 80% in oral cancer HSC-3 and Ca9-22 cell lines [106]. In a subsequent study, the authors showed that treatment with pomegranate extract caused cancer cells to suppress the G1 phase and increase their G2/M arrest, suggesting direct interference with their ability to proliferate. At the same time, a favorable regulation of apoptotic proteins was observed: levels of pro-apoptotic Bax protein and cleaved PARP increased, while anti-apoptotic proteins Bcl-2 and Bcl-xL decreased, favoring the programmed death of cancer cells [107]. The study also showed that pomegranate extract exerts its anti-tumor action by promoting the expression of antioxidant genes as well as impairing mitochondrial function in cancer cells. Overall, the studies indicate that pomegranate has a broad spectrum of anticancer activity against oral carcinoma, acting through multiple mechanisms including cell proliferation suppression, apoptosis, reduction in tumor invasion, oxidative stress, and DNA damage. These results underline the potential of pomegranate extract not only as a prevention strategy but also as a possible adjuvant in oral cancer treatments.

#### 3.2.12. Avocado

It is a tropical fruit with anti-tumor properties. Indeed, an in vitro study on human oral cancer cell lines demonstrated that avocado extracts are able to increase levels of reactive oxygen species (ROS) within oral cancer cells, thus triggering a process of programmed cell death (apoptosis). It is particularly relevant that this effect has been found to be selective, targeting cancer cells while healthy cells have not suffered significant damage. This suggests that the compounds in avocado could act as natural anti-cancer agents, able to induce oxidative stress in malignant cells without compromising the surrounding healthy tissues [108].

Similarly, the aliphatic acetogenins extracted from avocado are able to inhibit cancer cell proliferation in the human oral cancer cell line (83-01-82CA). In particular, it was shown that avocado chloroform extract D003, at non-apoptotic concentrations (20 μg/mL), and two of its components ((2S,4S)-2,4-dihydroxyheptadec-16-enyl acetate and (2S,4S)-2,4-dihydroxyheptadec-16-ynyl acetate) are able to inhibit the EGFR and the RAF/MEK/ERK1/2 signaling cascade in human oral carcinoma cells. The two compounds act through different mechanisms on this molecular pathway, suggesting a potential synergistic effect when combined at low concentrations, with an effective inhibition of cell proliferation [109].

#### 3.2.13. Vitamins

Another supplement with antioxidant and anti-cancer properties is *vitamin C*. The possible mechanisms of action for vitamin C in cancer prevention and control include: (1) counteracting inflammation and oxidative damage to DNA, involved in the development and progression of cancer; (2) cytotoxic effect due to the formation of hydrogen peroxide and ascorbate radical, which can induce cancer cell death; (3) promoting collagen synthesis and inhibiting hyaluronidase in order to hinder the spread of the tumor; (4) in synergy with other biological antioxidants and free radical scavengers, helping to break down the cascade of harmful free radicals [110,111,112,113]. Edefonti et al. analyzed the possible correlation between oral intake of vitamin C from fruit and vegetables and the risk of HNC. In this multicenter case-control study, the authors found that vitamin C intake is inversely associated with the risk of oral, pharyngeal, and laryngeal cancer (*p*-value < 0.05). Thus, increased intake of vitamin C from dietary sources is associated with a reduced risk of developing these cancers. Moreover, this study also showed that inverse associations between vitamin C intake and oral, pharyngeal, and laryngeal cancer risk were similar regardless of smoking status (never smokers, former smokers, and current smokers) and/or levels of alcohol consumption. This suggests that the protective effect of vitamin C on reducing the risk of these cancers is not dependent on smoking or alcohol, two known risk factors for HNC [114].

Bairati et al. carried out a randomized double-blinded and placebo-controlled clinical trial that evaluated the use of antioxidant vitamins (beta-carotene and alpha-tocopherol, or vitamin E) to prevent acute adverse effects of RT in HNC patients [115]. The 540 recruited patients were divided into two arms and received therapy throughout the RT and for three years thereafter: one group received daily doses of beta-carotene (30 mg/die) and vitamin E (400 IU/die); the other group received a placebo. The authors found that the antioxidant vitamin supplementation significantly reduced the post-RT adverse effects, most of all in the larynx [Odd Ratio OR 0.38]. However, patients taking beta-carotene and alpha-tocopherol had a higher risk of local cancer recurrence during the follow-up period compared to those in the placebo group [Hazard Ratio HR 1.37]. Therefore, the authors conclude that supplementation of these antioxidant vitamins can reduce the acute adverse effects of RT but at the cost of a reduced therapeutic efficacy of RT itself, probably because the antioxidants may protect cancer cells from radiation-induced oxidative damage, reducing the effectiveness of the treatment. Then, the same authors then conducted a further in-depth study on the use of antioxidants in patients with HNC undergoing RT to assess whether this supplementation also had an impact on overall mortality [116]. The results of the study confirmed an alarming fact: patients taking beta-carotene and vitamin E had a higher risk of mortality than those in the placebo group [HR 1.38]. The authors suggested that this may occur due to the ability of antioxidants to neutralize free radicals. Indeed, since RT destroys cancer cells by producing oxidative stress, the presence of high levels of beta-carotene and vitamin E may have reduced this effect, unintentionally protecting even malignant cells and allowing them to survive and proliferate. These findings are very relevant because they challenge the widespread belief that antioxidants are always beneficial to health. Actually, in the case of cancer patients undergoing RT, it seems that they can have a negative effect, reducing the effectiveness of the treatment and increasing the risk of mortality [117].

Vitamin D is a fat-soluble vitamin, which can be taken through the diet or produced by the body through exposure to UV-B rays from the sun. Vitamin D is not directly active but acts as a precursor of calcitriol (1α,25-dihydroxyvitamin D), the active hormonal form that performs numerous regulatory functions in the body, including controlling calcium metabolism and supporting the immune system. Vitamin D plays a key role in regulating the growth and spread of cancer cells, thanks to several mechanisms that contribute to its anti-tumor action. Vitamin D seems to play a protective role in the development and progression of HNC, as suggested by several preclinical studies conducted both in vitro and in vivo. The retrospective study by Grimm et al. examined the relationship between blood vitamin D levels and vitamin D receptor expression in patients with oral squamous cell carcinoma (OSCC) and precancerous lesions of the oral mucosa [118]. The results showed that patients with OSCC had very low serum vitamin D levels (<12.5 ng/mL), with a severe deficiency in 62% of cases; none of the patients had normal vitamin D levels. Moreover, in precancerous lesions, the VDR was found to be more expressed than in healthy tissue, indicating that in the early stages of disease, vitamin D may play a role in trying to counteract malignant transformation. However, in advanced OSCC tumors, the expression of the VDR was significantly reduced, suggesting that as the tumor progresses, malignant cells may lose their ability to respond to vitamin D, thus facilitating their uncontrolled growth. These results support the idea that vitamin D could play a preventive role by promoting apoptosis in precancerous cells. The authors hypothesize that the administration of vitamin D, both natural and synthetic forms, could have potential use in preventing tumor progression or even as a support to improve the effectiveness of radiotherapy and chemotherapy in the treatment of OSCC. Similar results have also been reported by Anand et al. in their prospective observational study [119]. Furthermore, HNSCC is characterized by an immune dysfunction induced by the cancer itself, which creates an inflammatory environment conducive to disease progression. The prospective placebo-controlled study by Kulbersh et al. investigated the potential of active vitamin D (1α,25-dihydroxyvitamin D) in modifying the tumor microenvironment in patients with HNSCC. In particular, the researchers focused on CD34(+) progenitor cells, known for their immunosuppressive properties, and their possible differentiation into mature dendritic cells, which are crucial to the activation of the immune response against cancer. In their study, patients with HNSCC were divided into two groups: one group received a treatment with active vitamin D for three weeks before surgery (4 µg of enteric 1,25(OH)₂D₃ administered daily for 3 consecutive days, followed by 4 days without treatment), while the other group did not receive any supplementation. The results showed that vitamin D treatment significantly reduced the number of CD34(+) cells within the tumor. At the same time, an increase in mature dendritic cells was observed, essential for the activation of an effective antitumor response. In addition, there was a decrease in immature dendritic cells, usually less efficient at counteracting tumor growth. These findings suggest that vitamin D could help to “rebalance” the tumor microenvironment, reducing the presence of immunosuppressive cells and promoting the activation of a more effective immune response against the tumor [120]. Bochen et al. reported that HNSCC patients with particularly low levels of vitamin D had an increased risk of lymph node metastases. Furthermore, patients with very low levels of vitamin D had a higher mortality rate than those with higher levels. Vitamin D deficiency also appeared to affect the immune microenvironment of the tumor, reducing the infiltration of T cells and natural killer (NK) cells, which play a key role in defending against the tumor. At the same time, an increase in M2 macrophages, which promote immunosuppression and tumor progression, was observed in patients with severe vitamin D deficiency. An interesting aspect of the study was the role of vitamin D supplementation. Indeed, when patients were given vitamin D, the activity of NK cells increased significantly, suggesting that an adequate level of vitamin D could improve the ability of the immune system to fight cancer. These results indicate that vitamin D deficiency is a common factor in patients with HNSCC and may have a negative impact on prognosis and immune response. The possibility that vitamin D supplementation may improve the effectiveness of cancer therapies is an issue that deserves further investigation, especially in the context of immunotherapeutic treatments [121]. In addition to the immunological role, vitamin D also seems to be linked to the nutritional conditions of patients with HNSCC, who often suffer from malnutrition before and after treatment. Weight loss and muscle depletion are aggravated by mucositis induced by RT. Some studies suggest that vitamin D supplementation may improve mucosal integrity in cancer patients with mucocutaneous toxicity [122]. Finally, insufficient dietary intake of vitamin D appears to be related to an increased risk of cancer recurrence [123]. The European Prospective Investigation into Cancer and Nutrition (EPIC) case-control study is one of the largest investigations on the link between vitamin D and HNSCC. Analyzing data from over 380,000 people in ten European countries, the researchers looked at the blood vitamin D levels of people who later developed HNSCC, comparing them with healthy people. The results showed that higher levels of vitamin D in blood were associated with a lower risk of developing HNSCC, with a 30% reduction in risk for each doubling of vitamin D levels. However, people with very low levels of vitamin D had a higher risk of death, but the same was also observed in those with excessively high levels, suggesting that optimal balance is crucial. A particularly protective effect of vitamin D has been found for cancers of the larynx and hypopharynx, where the risk was reduced by up to 58% with higher levels of vitamin D in the blood. However, the analysis revealed that vitamin D protection against HNSCC was only evident in smokers or former smokers, while in non-smokers vitamin D levels did not appear to have any impact on the risk of developing cancer [124]. Experiments on oral squamous cancer cell lines have shown that exposure to increasing concentrations of vitamin D reduces the proliferation of cancer cells. This effect is partly due to the activation of certain cell cycle inhibitors, such as p21, p18, and p27, proteins that block cell division and prevent cancer cells from continuing to grow unchecked [125,126,127]. Another mechanism by which vitamin D could counteract cancer is the suppression of telomerase activity, whereas telomerase is an enzyme that allows cancer cells to escape senescence, prolonging their survival and promoting the progression of cancer [128]. Furthermore, it promotes cell differentiation and stimulates DNA repair mechanisms, limiting the accumulation of genetic damage that could contribute to malignant transformation of cells. Meier et al. assessed the potential of vitamin D3 in preventing oral squamous cell carcinoma using the hamster buccal pouch model. The animals were exposed to a carcinogen and divided into two groups: one treated with vitamin D3 intraperitoneal injections (0.25 μg/kg 3 times per week), the other with a placebo. The results showed a significant protective effect: only 1 out of 10 hamsters in the treated group developed cancer, compared with 7 out of 10 in the control group (*p* < 0.01) [129]. Some genetic variants that affect vitamin D metabolism may have an impact on the risk and prognosis of head and neck squamous cell carcinoma (HNSCC). For example, patients with a specific vitamin D receptor variant (VDR FokI T/T), which reduces the biological activity of vitamin D, tend to have faster disease progression and shorter survival regardless of other factors such as age, smoking, or stage of the tumor [130]. Other genetic variants involved in vitamin D metabolism, such as those in the CYP2R1, CYP24A1, and vitamin D-binding protein genes, also appear to be related to blood vitamin D levels and overall survival of patients with HNSCC [131,132]. This suggests that the body’s ability to metabolize vitamin D may influence the course of the disease. In conclusion, the current literature suggests that vitamin D could play a key role in supporting the immune response, preventing nutritional complications, and potentially improving the prognosis of patients with HNSCC.

#### 3.2.14. Herbs in Traditional Medicine

Some herbs, such as Scrophularia buergeriana, Radix ophiopogonis, Radix rehmanniae, and Salvia miltiorrhiza, have proved to be a valuable supportive treatment for patients with nasopharyngeal cancer, improving clinical outcomes and reducing the side effects of conventional therapy (CT and RT). Several studies showed that the use of herbal medicine is associated with an improvement in tumor response (OR 1.67), indicating that patients treated with an integrated approach had a higher probability of obtaining a regression of the disease than those who received only chemotherapy-radiation therapy. In addition, the addition of medicinal herbs helped to reduce the side effects of conventional therapy (OR 0.48), such as nausea, fatigue, and myelosuppression, thus improving patients’ quality of life (OR 2.32) [133].

A prospective observational study conducted by Lim et al. investigated the effectiveness of herbal medicine in treating xerostomia in HNC patients undergoing RT. The study involved 42 patients: 28 received herbal treatment in addition to standard oncological surveillance, while 14 received only standard surveillance. The most used herbs in treatment included Wu Mei, San Qi, and Tian Hua Fen, while the commonly prescribed herbal formulas were Sha Shen Mai Dong Tang, Liu Wei Di Huang Wan, and Gan Lu Yin. After six months, the herbal treatment group had a significant improvement in stimulated salivary flow rate (*p* < 0.05). Also, these patients reported an improvement in their quality of life (Head and Neck Cancer-Specific Quality of Life Questionnaire), particularly regarding the ability to speak and eat and a reduced head and neck pain (*p* < 0.05) [134]. These findings suggest that herbal medicine may offer benefits in the treatment of xerostomia and improve quality of life in patients with head and neck cancers post-radiation therapy [135].

Among the many herbs used in traditional medicine, Malva sylvestris and Alcea digitata are distinguished by their beneficial properties, particularly in the treatment of xerostomia. Malva sylvestris, used for centuries for its soothing, emollient, and anti-inflammatory properties, is rich in mucilages, polysaccharidic compounds that can form a protective film on the mucous membranes, reducing irritation and improving hydration of the oral cavity. Alcea digitata, another plant in the Malvaceae family, is characterized by a high content of antioxidant and mucilage compounds [136]. In 2016, Ameri et al. evaluated the effectiveness of an herbal compound with Malva sylvestris and Alcea digitata in treating xerostomia in HNC patients. In this study, 75 patients were randomly assigned to two groups: one received a compound based on Malva sylvestris and Alcea digitized powder for 4 weeks, while the other used artificial saliva for the same period. The results showed that the group treated with herbal compound reported a significant reduction in the severity of xerostomia compared to the control group (*p* < 0.05), suggesting the effectiveness of herbal treatment in improving salivary function in these patients [137]. These results were confirmed by the randomized double-arm study conducted by Heydarirad et al. [138].

Holy Basil (Ocimum sanctum) is a medicinal herb from Thailand, rich in flavonoids and phenolics, with antioxidant and anti-tumoral effects. An in vitro study conducted on SCC HNC cell lines showed that extracts from Ocinum sanctum leaves can decrease the activity of MMP-2 and MMP-9 metalloproteinases, thus significantly reducing cancer cell invasion (*p* < 0.05) [139]. The inhibiting action on metalloproteinases suggests that this plant could interfere with biological processes that promote tumor metastasization. Furthermore, Ocimum sanctum extract has cytotoxic activity against the oral carcinoma Ca9-22 cell line, suggesting its potential as a natural chemotherapy agent [140].

#### 3.2.15. Chinese Herbal Medicine

Today, Chinese herbal medicine is now widely recognized as a valid adjuvant treatment for cancer that can strengthen the immune system, contributing to improved patient prognosis [141]. The mechanisms of action of this alternative medicine are not clear. Probably, natural compounds from these herbs can impair angiogenesis and the growth of cancer cells by regulating the expression of nuclear factor κB (NF-KB) [142]. Furthermore, Chinese herbs can mitigate the adverse effects of chemotherapeutic drugs; however, the herbal compounds may impair anti-cancer effects by modulating enzyme expression [143]. Therefore, the selection of Chinese herbal medicine as an adjuvant therapy to CT must be carefully considered to avoid potential interference and a reduction in the chemotherapeutic effect. In particular, a 2023 review analyzing studies about Chinese herbal medicine in oral cancer found that Chinese herbal medicines may suppress cancer cell growth by triggering the intrinsic apoptotic pathway [144]. This mechanism of action is similar to the action of chemotherapy drugs used for oral carcinoma, namely by inducing apoptosis of cancer cells. Specifically, some studies have shown that among Chinese medicinal herbs, flavonoids have the most targeted anti-tumor action against oral cancers [145,146]. The retrospective study conducted by Wang et al. offers an interesting perspective on the possible impact of Chinese Herbal Medicine in improving the survival of patients with advanced nasopharyngeal cancer. The analysis was based on clinical data of Taiwanese patients, comparing those who received conventional treatments with and without the support of Chinese phytotherapy. The results showed that patients who have integrated Chinese Herbal Medicine into their therapeutic path have reported a reduction in mortality risk, suggesting a potential role of these remedies in supporting standard cancer therapies. This effect was particularly evident in the most advanced cases (stage III and IV tumors), where long-term survival is often compromised [147]. Similarly, Liu et al. explored the effect of Shengmai Yin on the state of DNA methylation in nasopharyngeal carcinoma cells and their radioresistive variants. The research revealed that radio-resistant cells exhibited widespread genomic hypomethylation, suggesting a correlation between DNA methylation and resistance to RT. The Shengmai Yin showed the ability to partially restore the original methylation state, thus increasing the sensitivity of cells to RT. In particular, the Tenascina-C gene, overexpressed in radio-resistant cells, has been downregulated after treatment with Shengmai Yin [148]. 

The systematic review conducted by Nik Nabil et al. analyzed the efficacy and safety of traditional Chinese medicine in the management of radiotherapy-induced xerostomia in patients affected by HNC. From the results analyzed, Chinese herbs have shown significant potential in improving post-radiation xerostomia, not only alleviating the feeling of dryness but also promoting the recovery of salivary function. The effectiveness of Chinese phytotherapy in post-radiotherapy xerostomia is based on several mechanisms of action. The first is the neuroprotective effect on the salivary glands, where herbs such as Panax ginseng help preserve nerves damaged by RT, thus stimulating increased production of saliva. Another key mechanism is the antioxidant action provided by plants such as Ophiopogon japonicus and Schisandra chinensis, which counteract oxidative stress responsible for damage to salivary glands and promote their regeneration. The regulation of inflammation is an additional benefit offered by these herbs since they reduce the inflammatory response in the irradiated glands, facilitating the recovery of their functionality. Finally, many herbal formulas improve blood circulation and the hydration of the mucous membranes, promoting an increase in salivary flow and reducing the feeling of dryness, thus improving patients’ comfort [149].

### 3.3. Cannabinoids

Studies about cannabinoids show conflicting results regarding the association between cannabis use and risk or benefits in the case of HNC. Indeed, Whyte et al. conducted an in vitro study on oral cells demonstrating that cannabinoids inhibit mitochondrial respiration, leading to a reduction in the production of Adenosine Triphosphate (ATP), the main source of cellular energy, thus affecting the energy metabolism of cancer cells. This effect may represent a mechanism by which cannabinoids exert an anti-tumor action, inducing metabolic stress that hinders the growth and survival of malignant cells [150]. On the contrary, in their cohort study, Gallagher et al. reported that patients with cannabis-related disorders had an increased risk of developing HNC compared to those who did not use cannabis (Relative Risk RR 3.49). In particular, the risk of oral cancer is more than double that of non-users (RR 2.51); for oropharyngeal cancer, the risk is almost five times higher (4.90). The most impressive result concerns laryngeal cancer, where the risk is over eight times higher than those who do not use cannabis (RR 8.39). These findings suggest that heavy and chronic cannabis use could have similar or even worse harmful effects than tobacco, especially on the larynx. This may be due to the way in which cannabis is consumed, often without filters and with a deeper and longer inhalation, exposing the respiratory tract to high temperatures and potentially harmful substances [10].

### 3.4. Mind-Body Therapy

Musculoskeletal impairment (MSI) is a complication of HNC therapy that, although very important, is often underestimated. It can lead to numerous problems, including pain, altered posture, and limitations in basic movements of the neck and jaw. In addition, MSI can also have a significant impact on the psychological level, contributing to disorders such as anxiety and discomfort related to body image. In addition to this, there is an increase in health care costs and a possible reduction in working capacity, with repercussions on patients’ quality of life [151,152]. 

Yoga has its origins in India; it is increasingly used in the West for the treatment of various pathologies since the 70′s and is currently recognized by the National Institutes of Health as a practice belonging to Complementary and Alternative Medicine [153]. In the oncology context, most yoga studies highlighted significant benefits in managing symptoms related to the disease, improving psychological well-being, and overall quality of life [154,155]. The basic elements of yoga practice include postures, controlled breathing, relaxation, and meditation. In particular, a growing number of research studies show how yoga can help reduce stress and improve systemic symptoms such as pain, fatigue, anxiety, depression, and insomnia [156,157]. Adair et al. evaluated the feasibility and preliminary effectiveness of a personalized hatha yoga program for 40 HNC survivors. Participants were randomly assigned to an 8-week yoga intervention or a waiting list control group. The Hatha yoga program included postures (asanas), breathing exercises (pranayama), meditation, and relaxation techniques. The results showed that the yoga program was feasible and safe, with high recruitment and membership rates, no adverse events, and high satisfaction of participants. In addition, significant improvements were observed in the range of shoulder movement, reduction in pain (*p* < 0.001), and anxiety (*p* = 0.015) in participants in the yoga group compared to the control group. Thus, the study demonstrated that a personalized hatha yoga program is not only feasible but also potentially effective in improving physical function and psychological well-being in HNC survivors [158]. In 2018, Anand et al. explored the use of yoga as an integrative approach in the prevention and treatment of oral cancer. Indeed, oral cancer is often associated with risk factors such as obesity and may have an inverse relationship to neurodegenerative disorders. The study suggests that yoga could positively influence cellular biomarkers such as nerve growth factor (NGF), TNF-α, and interleukin-6 (IL-6), which are involved in the progression of oral cancer, obesity, and neurodegenerative disorders. The authors propose that yoga could help modulate the expression of these molecules, offering a potential benefit in preventing and treating oral cancer [159]. While Milbury et al. examined the feasibility and preliminary effectiveness of a couple yoga program (dyadic yoga) for HNC patients undergoing chemoradiotherapy and their family caregivers [160]. The study involved 37 couples; participants were randomly assigned to two groups: one followed a yoga program structured in 15 sessions, parallel to the therapeutic path of the patient, while the other control group did not participate in the yoga program. Results showed that the program was feasible and highly appreciated by participants. In patients who practiced yoga, there was a significant reduction in symptoms related to the disease and treatment, with an improvement in quality of life compared to the control group. In addition, these patients were less likely to be hospitalized (OR 3.00), to need first aid, or to require a feeding tube during and after therapy (OR 1.78). In summary, the study suggests that couple yoga could be a valuable support for cancer patients under treatment, improving their physical and emotional well-being while relieving the burden of caregivers.

In addition to progressive muscle relaxation and guided imagination, calligraphy handwriting can also have a therapeutic effect on patients with cancer. This is what the authors evaluated in their randomized controlled trial on patients suffering from nasopharyngeal carcinoma [161]. They demonstrated that both the practice of calligraphy and relaxation training had positive effects. In particular, calligraphy training has led to a gradual reduction in systolic blood pressure (*p* = 0.007) and respiratory rate over time (*p* = 0.000), although with a lesser effect than relaxation. In addition, relaxation has been shown to be effective in reducing insomnia and improving mood disorders, while calligraphy has increased the level of concentration (*p* = 0.032) and improved mood disturbance. 

**Figure 2:**
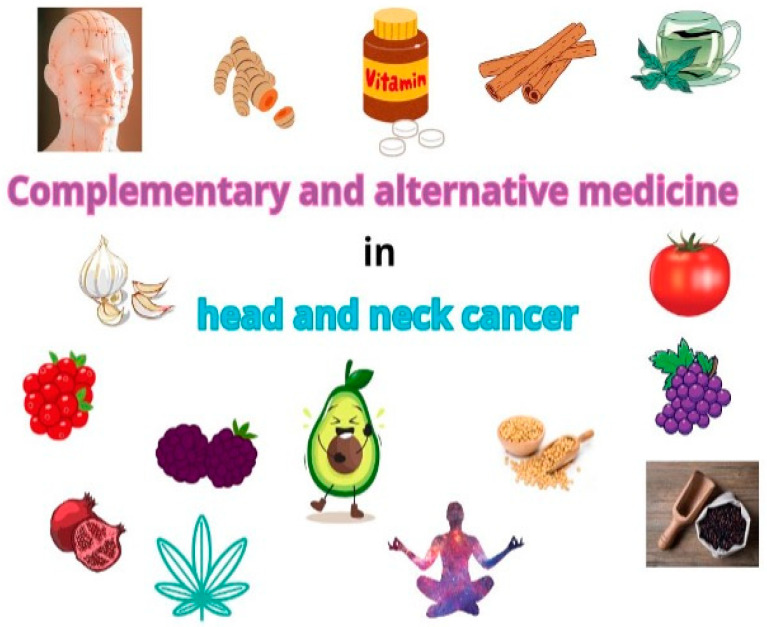
Complementary therapies in head and neck cancer: acupuncture, curcumin, black raspberry, cranberry, green tea, tomato, soybean, black rice, cinnamon, garlic, grape, pomegranate, avocado, vitamins, cannabinoids, yoga.

Another randomized controlled study was conducted on patients with nasopharyngeal carcinoma undergoing chemoradiotherapy. In this case, the authors examined the effect of Tai Chi exercise on cancer-related fatigue. The results of the study showed that patients who followed the Tai Chi program reported lower levels of fatigue overall than the control group, with significant improvements in both physical and emotional fatigue (*p* > 0.01). In addition, these patients reported an increased sense of vigor and energy, suggesting that the exercise has helped not only to reduce fatigue but also to improve general well-being. Improving the regulation of the autonomic nervous system could be one of the mechanisms by which this practice helps to counteract fatigue, offering patients both physical and mental support during the treatment [162] (Table 1) (Figure 2).

## 4. Discussion

Interest in alternative therapies for the treatment of HNC has grown significantly in recent years, both for research into less invasive and more tolerable therapeutic strategies and the need to mitigate the side effects of conventional therapies. However, the efficacy and safety of many of these therapies remain a subject of debate, and the scientific evidence supporting them is often limited or controversial. Although some practices have shown beneficial effects on specific symptoms, their application in clinical practice remains uncertain due to variability of results and the lack of standardized protocols.

Acupuncture, one of the most studied alternative therapies, has been shown to be effective in improving symptoms such as xerostomia and post-laryngectomy dysphagia. Some clinical studies have reported a significant improvement in the quality of life of treated patients, with a reduction in oral dryness and an improvement in swallowing [28,30,31,32,33,34,35,36,44]. Notably, the studies included in this review were clinical trials, mostly single-blinded and placebo-controlled, and were approved by local ethics committees. However, there are still doubts about the reproducibility of the results and their effectiveness compared to placebo treatments. In particular, about xerostomia, the available studies show that the beneficial effects of acupuncture tend to be limited in time, with improvements only until shortly after the treatment period (4–6 months). Only one study has documented a prolonged benefit up to one year after the end of therapy [30]. Therefore, further research needs to investigate the long-term efficacy of acupuncture for xerostomia. Moreover, the efficacy of acupuncture in these studies was assessed mainly through questionnaires and tests administered to patients, making the assessment of therapeutic benefit inherently subjective. This leads to a certain variability in the interpretation of results. It is important to note that a statistically significant result does not necessarily correspond to a real clinical improvement perceived by the patient. For the Xerostomia Questionnaire, the studies included in this review consider scores above 30 as indicative of clinically significant xerostomia. In addition, two out of three refer to a Minimal Clinically Important Difference equal to 10 points, a threshold useful for determining whether the observed variation is perceived by the patient as a real improvement. The joint consideration of these two parameters absolute value and clinically relevant variation is essential for a correct interpretation of the results. In addition, the lack of standardization in acupuncture techniques used in different studies makes it difficult to assess its effectiveness consistently [34,41].

Phytotherapy includes several natural compounds with potential antitumor and symptomatic effects. Substances such as turmeric, resveratrol, tomato, green tea, and various plant extracts have shown in vitro and in animal models antioxidant, anti-inflammatory, and pro-apoptotic properties. Studies have shown reduced tumor growth and increased sensitivity of cancer cells to RT and CT [48,49,50,51,52,76,77,78,79,80,81,82,84,88,89,109]. However, their clinical efficacy requires further validation with large-scale studies. In addition, the bioavailability of many of these substances is a significant limitation to their clinical application, making it necessary to research more effective formulations [70,71,72,74]. It should also be noted that, as in the case of black raspberry, cranberry, and cinnamon, most studies have been conducted in vitro or on animal models in vivo, and therefore their application and actual efficacy in human oral carcinoma remain largely unknown. In addition, about lycopene, a compound present in tomatoes, several randomized studies have shown its anti-cancer efficacy in oral carcinoma. Although animal model studies [86,87] suggest that the efficacy of lycopene is not dose-dependent, evidence from in vivo human studies suggests otherwise: efficacy appears to increase with dose, with more significant results observed at 8 mg per day compared to lower doses [88]. In addition, it has been observed that reduced blood levels of lycopene may be associated with an increased risk of cancer-related mortality [89]. In conclusion, since most studies have been conducted in vitro or on animal models, and the clinical trials on humans are still limited, the actual effectiveness of these substances, as well as the optimal dose and potential side effects, remain largely unknown.

Vitamins and antioxidants are an area of particular interest, with some evidence suggesting a protective effect against HNC [114,118,119,120]. However, clinical studies have shown possible adverse effects: the intake of beta-carotene and vitamin E, for example, may increase the risk of local relapse in patients undergoing RT, probably reducing the effectiveness of treatment-induced oxidative stress [115,116,117]. Interest in vitamin D in the context of HNC has increased in recent years, thanks to several pieces of evidence suggesting that it may have a protective role in both preventing and controlling the disease. Several preclinical studies have shown that vitamin D can reduce the proliferation of cancer cells, promote their differentiation, promote apoptosis, and stimulate DNA repair. It also appears to have a significant influence on the tumor microenvironment, helping to strengthen the immune response against malignant cells. However, despite these promising results, the efficacy of vitamin D in cancer patients has yet to be clarified. Much of the evidence available comes from in vitro or animal model studies, while human clinical trials are still limited. In addition, the effective and safe dosage is not well defined: in some animal models, for example, doses considered useful have caused important side effects such as hypercalcemia and weight loss [163]. Hence the interest in the development of vitamin D analogues, theoretically less toxic but still little studied in humans. Another element to consider is individual differences in vitamin D response, linked to genetic variants that affect metabolism and receptor activity. Finally, it has been observed that in advanced tumors the expression of the vitamin D receptor is reduced, which could limit its effectiveness in the clinically most critical cases. In summary, while vitamin D appears to be a promising agent that can influence various aspects of cancer and the immune system, important questions remain about its real clinical usefulness, optimal dose, safety, and appropriate time for possible administration. Indeed, in their study, Meier et al. highlighted how vitamin D was administered simultaneously with the carcinogen, a condition far removed from the clinical reality of patients with HNC, who have a long history of exposure to risk factors such as smoking and alcohol [112].

Traditional Chinese medicine, including herbal medicines and techniques such as Qi Gong, is now increasingly recognized as an adjuvant treatment for cancer, thanks to its ability to modulate the immune system and improve tolerance to conventional therapies. However, its mechanisms of action are not yet entirely clear, and some herbs could interfere with the efficacy of chemotherapy, which is why their use should be evaluated with caution. Recent studies suggest a direct antitumor effect, mainly through the activation of apoptosis, with an important role of flavonoids. Some studies also indicate a possible benefit in terms of survival in patients with advanced tumors and an increased sensitivity to radiotherapy due to epigenetic modulation. In addition, the use of Chinese herbs has shown promise in the treatment of post-radiotherapy xerostomia, thanks to neuroprotective, antioxidant, and anti-inflammatory effects that promote the regeneration of salivary glands and improve patients’ quality of life [133,134,140,144,145,146]. 

Cannabinoids are a controversial topic. While some studies suggest an inhibitory effect on tumor growth and a palliative action to control pain and nausea [150], chronic cannabis use has been associated with an increased risk of HNC, probably due to combustion and inhaled toxic substances. In addition, the mechanism of action of cannabinoids in the oncology context is not yet well defined, and further studies are needed to better understand the potential risks and benefits of these compounds [10].

Finally, the integration of mind-body practices such as yoga, tai chi, or even calligraphy is emerging as a useful option in the context of treating patients with head and neck cancers. In addition to the documented physical benefits, such as reducing pain and improving mobility, these techniques also seem to have a positive impact on psychological well-being, helping to reduce anxiety, insomnia, and fatigue related to therapies. Recent studies have shown that structured interventions, even in the active phase of treatment, are not only feasible and safe but potentially effective in improving the quality of life of patients and their caregivers. These approaches, while not replacing conventional therapies, can be a significant support for better dealing with the side effects and psychophysical relapses of disease. However, their role in directly modifying tumor progression remains to be clarified, and further research is needed to define their oncological efficacy [158,159,160,161]. 

Overall, although some alternative therapies have shown potential benefits, the lack of robust clinical trials and standardized protocols makes their integration into conventional cancer practice difficult. Another important limitation is that most available studies are not double-blinded, and the evaluation of therapeutic efficacy is often based solely on subjective tools such as questionnaires and patient-reported outcomes. This introduces a potential bias in the interpretation of results. Moreover, the type of questionnaire used for quality-of-life assessment is specified only in a few studies [61,139], and the reference value of the Minimal Clinically Important Difference is not reported. This lack limits the possibility of accurately interpreting the real clinical relevance of the observed differences, since statistical significance alone is not sufficient to determine a concrete impact on patients’ subjective perception. The use of such therapies should always be discussed with the oncology team to avoid possible negative interactions with conventional treatments. It is therefore crucial that alternative therapies are evaluated with the same scientific rigor as traditional therapies, ensuring both the safety of cancer patients and the reliability of their potential clinical benefits.

## 5. Conclusions 

The analysis shows that alternative therapies may play a complementary role in the treatment of head and neck cancer but should never be considered as substitutes for conventional therapies. Some strategies, such as acupuncture and herbal medicine, have shown beneficial effects on specific symptoms, while others, such as cannabinoids and antioxidants, raise safety issues and need further investigation. The integration of these therapies should be performed with caution and under the supervision of qualified health professionals. 

The future of research should focus on conducting rigorous clinical trials to assess the efficacy, safety, and potential interactions of these therapies with standard cancer treatments. In addition, the development of innovative formulations to improve the bioavailability of certain natural compounds could be a step towards maximizing their benefits.

Finally, although medical science continues to make progress in improving cancer care, prevention remains a crucial aspect. A healthy lifestyle, based on a balanced diet and regular physical activity, can contribute significantly to reducing the risk of developing cancer. As the saying goes, “one apple a day takes the doctor away”, but only if accompanied by awareness and prudence in the use of alternative therapies.

## Figures and Tables

**Table 1:** Pro and cons of complementary therapy in head and neck cancer.

Therapy	Evaluated in Clinical Trials	Mechanism of Action	Benefits	Limitations/Risks	Future Perspectives
Acupuncture	Yes–randomized controlled trials	Neural stimulation, vascular modulation, activation of swallowing reflex	Improvement of xerostomia and dysphagia	Doubts about reproducibility and standardization	Better standardization of application techniques
Phytotherapy	Partially (curcumin, green tea, lycopene)	Antioxidant, anti-inflammatory, pro-apoptotic	Tumor growth inhibition, mucositis reduction	Low bioavailability, need for clinical validation	Improved formulations to increase bioavailability
Vitamins and antioxidants	Yes	Oxidative stress modulation, apoptosis, immune support	Potential protective effect, but controversial	Possible increased risk of relapse with some antioxidants (vitamin E)	More rigorous studies to assess risks and benefits
Cannabinoids	Limited (mostly pre-clinical, observational)	Inhibition of cell metabolism, palliative effects	Possible palliative action, but controversial	Chronic use associated with increased head and neck cancer risk	Investigation of the mechanism of action for oncology use
Traditional Chinese Medicine	Some clinical data (e.g., xerostomia)	Immunomodulation, antioxidant and neuroprotective effects	Improving quality of life, reduction in side effects	Possible negative interactions with radiotherapy/chemotherapy	Safety and efficacy studies for oncology integration
Mind-body therapies	Yes–yoga, Tai Chi, relaxation (randomized controlled trials)	Modulation of stress hormones; autonomic balance	Improving quality of life, reducing stress and fatigue	Uncertain role in tumor progression	Further research to define its role in cancer therapy

## Data Availability

Publicly available datasets were analyzed in this study. These data.can be found here: PubMed (https://pubmed.ncbi.nlm.nih.gov, accessed on 7 January 2025) and Scopus (https://www.scopus.com, accessed on 7 January 2025).

## Abbreviations

HNCs	Head and Neck Cancers
SCC	Squamous Cell Carcinoma
OC	Oral Cancer
OPC	Oropharyngeal Cancer
HPV	Human Papillomavirus
TNM	Tumor-Node-Metastasis
RT	Radiotherapy
CT	Chemotherapy
WHO	World Health Organization
FDA	Foods And Drug Administration
COX-2	Cyclooxygenase-2
ROS	Reactive Oxygen Species
EMT	Epithelium-Mesenchymal Transition
TME	Tumor Microenvironment
CAFs	Cancer-Associated Fibroblasts
PBM	Photobiomodulation
POCP	Preventive Oral Care Program
MAO	Monoamine Oxidase
TNF	Tumor Necrosis Factor
PCNA	Proliferating Cell Nuclear Antigen
DMBA	7,12-Dimethylbenzathracene
GPx	Glutathione Peroxidase
CAT	Catalase
SOD	Superoxide Dismutase
OR	Odd Ratio
HR	Hazard Ratio
VDR	Vitamin D Receptor
HNSCC	Head And Neck Squamous Cell Carcinoma
OSCC	Oral Squamous Cell Carcinoma
NK	Natural Killer
EPIC	European Prospective Investigation Into Cancer And Nutrition
ATP	Adenosine Triphosphate
RR	Relative Risk
MSI	Musculoskeletal Impairment
NGF	Nerve Growth Factor
IL-6	Interleukin-6
